# Supplementary material for: Application of Seaweed Generates Changes in the Substrate and Stimulates the Growth of Tomato Plants
Source: Plants (Basel). 2023 Mar 31;12(7):1520. doi: 10.3390/plants12071520 (PMC10096834; doi:10.3390/plants12071520)
Supplement: Supplementary file 1 [file plants-12-01520-s001.zip › plants-2276501-supplementary.pdf]

Table S1. Percentage of biomass remaining and N remaining in the decomposed dry biomass and N released to the substrate.

Time (days)	Remaining dry mass	Remaining N	Mineralized N
0	100± 0.00a	100± 0.00a	0± 0.00f
7	39.54± 1.10b	79.85± 1.03b	20.14± 2.32e
14	27.38± 2.21c	74.01± 1.01b	25.98± 1.21d
21	20.30± 2.21d	65.79± 2.11c	34.2± 1.012c
28	19.24± 3.41d	54.98± 2.101d	45.01± 2.01b
35	18.57± 3.21d	54.46± 3.01d	45.53± 2.02b
42	10.38± 2.21e	29.6± 1.11e	70.39± 3.05a
49	8.59± 2.11e	-	-
56	6.6± 2.016e	-	-

nd = not determined. Different letters (a–e) within the rows indicate significant differences of each treatment in com-parison to the control (untreated substrate), based on the Holm–Sidak means comparison test ($p < 0.05$).

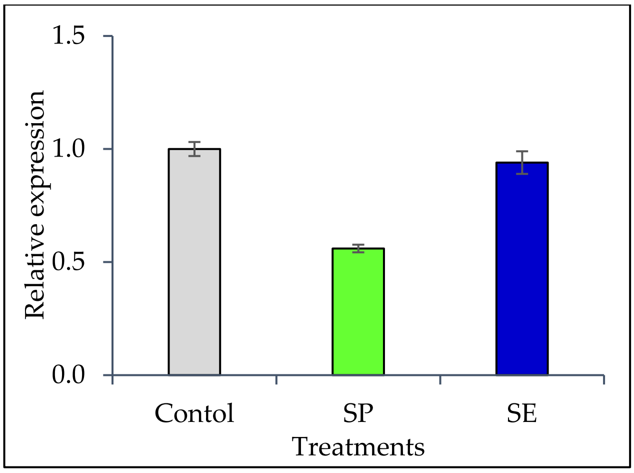

Figure S1. Expression analysis of the SlHB7 gene related to the response to salinity stress in tomato. Transcript levels analyzed in pooled leaves of three 45-day-old tomato plants treated with seaweed powder (SP) or seaweed extract (SE) from *Ulva ohnoi*. Values represent mean ± standard deviation (n=3).
